# Supplementary material for: Susceptibility of Aerococcus urinae and Aerococcus sanguinicola to Standard Antibiotics and to Nitroxoline
Source: Microbiol Spectr. 2023 Feb 27;11(2):e02763-22. doi: 10.1128/spectrum.02763-22 (PMC10100651; doi:10.1128/spectrum.02763-22)
Supplement: Supplemental file 1 — Fig. S1 and S2. Download spectrum.02763-22-s0001.pdf, PDF file, 0.6 MB [file spectrum.02763-22-s0001.pdf]

Susceptibility of *Aerococcus urinae* and *Aerococcus sanguinicola* to standard antibiotics and to nitroxoline

Aysel Ahmadzada, Frieder Fuchs and Axel Hamprecht

Supplementary data

Aerococcus urinae

n=166 r=-0,5; p<0,001

y = -1,4662x + 35,313

MIC (mg/L)	12	13	14	15	16	17	18	19	20	21	22	23	24	25	26	27	28	29	30
64	1			1															
32		2																	
16					1														
8												1							
4											1	1							
2								1	4	8	7	5	3	1					
1								3	9	20	16	18	13	7	8	4	4	1	2
0,5							1	1	2	2	4	2	2	3	3	1			
0,25												1		1	1				

inhibition zone diameter (mm)

FIG S1 Scattergram of nitroxoline MICs versus zone diameters (n= 166) for *A. urinae;* scattered lines indicate EUCAST breakpoints for *E. coli* (Susceptible: ≤ 16 mg/L or inhibition zone ≥15 mm).

Aerococcus sanguinicola

MIC (mg/L)	6	7	8	9	10	11	12	13	14	15
254										
128					1	1		2		
64	1	1	1	1	1	1	2	2		
32		2			1	1				
16										

inhibition zone diameter (mm)

*n=18 r=0,4; *p=0,103*
y = 5,6384x + 13,474

FIG S2 Scattergram of nitroxoline MICs versus zone diameters (n= 18) for *A. sanguinicola;* scattered lines indicate EUCAST breakpoints for *E. coli* (Susceptible: ≤ 16 mg/L or inhibition zone ≥15 mm)
